# Accuracy of in vitro mandibular volumetric measurements from CBCT of different voxel sizes with different segmentation threshold settings

**DOI:** 10.1186/s12903-019-0891-5

**Published:** 2019-09-04

**Authors:** Ting Dong, Lunguo Xia, Chenglin Cai, Lingjun Yuan, Niansong Ye, Bing Fang

**Affiliations:** Department of Orthodontics, Ninth People’s Hospital Affiliated to Shanghai Jiao Tong University, School of Medicine, No. 639 Zhizaoju Road, Shanghai, China

**Keywords:** Cone-beam computed tomography (CBCT), Voxel size, Hounsfield unit threshold, Volumetric measurement

## Abstract

**Background:**

To determine the accuracy of volumetric measurements of the mandible in vitro by cone-beam computed tomography (CBCT) and to analyze the influence of voxel sizes and segmentation threshold settings on it.

**Methods:**

The samples were obtained from pig mandibles and scanned with 4 voxel sizes: .125 mm, .20 mm, .30 mm, and .40 mm. The minimum segmentation thresholds in Hounsfield units (HU) were set as 0, 100, 200, 300, and 400, respectively, for each voxel size for 3D reconstruction. Laser scanning as the reference, the volumes of each CBCT scanning, the mean iterative distances of superimposition and total positive and negative deviations were recorded and compared.

**Results:**

The volumes of CBCT-scan deviated from those of laser-scan by + 7.67% to − 3.05% with different HU and voxel sizes. The deviation increased with the voxel size. There was a more suitable minimum HU threshold of segmentation (HU100 for .125 mm, 200 for .20 mm, 300 for .30 mm, and 400 for .40 mm) for each voxel size.

**Conclusions:**

Voxel sizes and Hounsfield unit thresholds influence the accuracy of volumetric measurements in CBCT scanning. The volume increase with the voxel size, and different voxel sizes correspond to different optimal Hounsfield unit thresholds.

## Background

Three-dimensional (3D) reconstruction of maxillofacial structures with cone-beam computed tomography (CBCT) has been widely applied in orthodontics [1], oral and maxillofacial surgery [2], oral implantology, and other fields. The inaccuracy of volumetric measurement in CBCT reconstruction may have important clinical influence.

First, such inaccuracy will influence diagnosis, such as 3D cephalometric analysis, which is necessary to identify cephalometric landmarks on 3D volumetric surfaces; further, the accuracy of maxillofacial reconstruction affects the precision of landmark identification and measurement analysis [3]. Recently, many scholars focused automated 3D cephalometric landmarking on CBCT volumes, where landmarks that have been located are directly annotated in volume voxels. Montúfar et al. [4] investigated a model-based algorithm for automatic landmarking in CBCT volume and scored a 3.6-mm error for 18 landmarks. Gupta et al. [5] reported an average error of 2.01 mm for 20 landmarks, with 64.67% of the landmarks in a range of 0 to 2 mm, 82.67% from 0 to 3 mm, and 90.33% from 0 to 4 mm. The deviations in 3D-reconstructed volume will create unavoidable error in landmark positioning.

Second, the inaccuracy of volumetric measurement will influence creation of the bone-supported guide template for use in orthognathic surgery, which is indicated for the treatment of significant skeletal malocclusions and facial dysmorphosis. Accurate volumetric measurements and the surface profiles of jaws play an important role in the design of jaw movement and contour trimming. Ye et al. [6] developed a method for a computer-image-guided surgical template for the navigation of Mandibular Angle Ostectomy. The template was obtained from the reconstructed mandibular model, so the volume of the mandibular model would doubtless affect the precision of the template. Various surgical devices have been developed to connect intact anatomic structures other than the maxillary and mandibular jaws, which are displaced during surgery. Lee et al. [7] examined the precision of a CAD/CAM facebow-based surgical guide template by comparing it with a bite wafer and found significant intergroup differences in lateral error compared with the absolute values of the 3D linear distance. Thus, it can be seen that artefacts of 3D volumetric surface can degrade the precision of orthognathic surgery design.

Third, the inaccuracy of volumetric measurement can influence superimposition and comparison before and after surgery, such as with bone augmentation and orthognathic surgery. CBCT and 3D reconstruction can be used to assess the efficiency of bone augmentation by aiding in the evaluation of the osteogenesis of grafting materials, and the accuracy of volumetric measurement plays a key role here. Lo et al. [8] investigated the relationship between soft- and hard-tissue changes after orthognathic surgery. Pre- and postoperative CBCT images were superimposed by the surface registration method, and the volumetric differences of each region were used to estimate the average movement. If the volumes of craniofacial structures before and after surgery disagree with each other, the reconstructed models cannot be precisely superimposed, especially affecting the accuracy and stability evaluation of orthognathic surgery. For a larger structure that requires more than one scan due to the limited field of view (FOV) of CBCT, the images of the two scans cannot be well-integrated, as a result of different volumetric errors.

However, there has been limited research on the volumetric error of 3D reconstruction with CBCT. As we know, the quality of images obtained by CBCT depends on many acquisition parameters, such as tube voltage, tube current, the field of view (FOV), the Hounsfield unit (HU) threshold of segmentation, and voxel size [9]. The first aim of this study was to compare the accuracy of volumetric measurement of CBCT scans. The second aim was to evaluate the effects of voxel sizes and HU thresholds on volumetric measurements and to assess optimal segment thresholds for different voxel sizes of CBCT volumetric measurement. In considering radiation, we chose pig mandibles for preliminary in vitro exploration.

## Methods

For this study, we obtained pig mandibles from a butcher and removed the soft tissues, teeth, and alveolar bone from the mandibles. This study was approved by the Institutional Review Board of Shanghai Ninth People’s Hospital affiliated to Shanghai Jiao Tong University, School of Medicine (2018–87-T78). For the FOV we used, we cut the pig mandibles into several small pieces and included 24 pieces in the study. Because a laser scan is a surface scan, we filled the cavities on the mandibular surfaces with a mixture of plaster and bone meal. The samples were coated with Arti-Spray BK-285 powder (Dr Jean Bausch, Köln, Germany) to facilitate laser scanning. The laser scan was performed with a 3Shape scanner (R700, 3Shape, Copenhagen, Denmark), which can obtain surface models in the form of a “point cloud” format with an accuracy of 20 mm and produce a stereolithography (STL) digital representation of the physical object [10]. The laser-scanned models were exported as STL format files to Geomagic Control software (Geomagic Control 2015.1.1; 3D Systems, Rock Hill, SC, USA) and were regarded as the reference.

A Hounsfield unit threshold must be calibrated before use due to variations among manufacturers. Therefore, we adopted the method described by Ye et al. [10] and scanned wax and a cup of water with the CBCT machine (KaVo Dental, Biberach, Germany) according to the manufacturer’s instructions. The means and standard deviations of the thresholds of water, air, and wax were calculated in Hounsfield units with eXamVisionQ software (KaVo Dental).

**Fig. 1:**
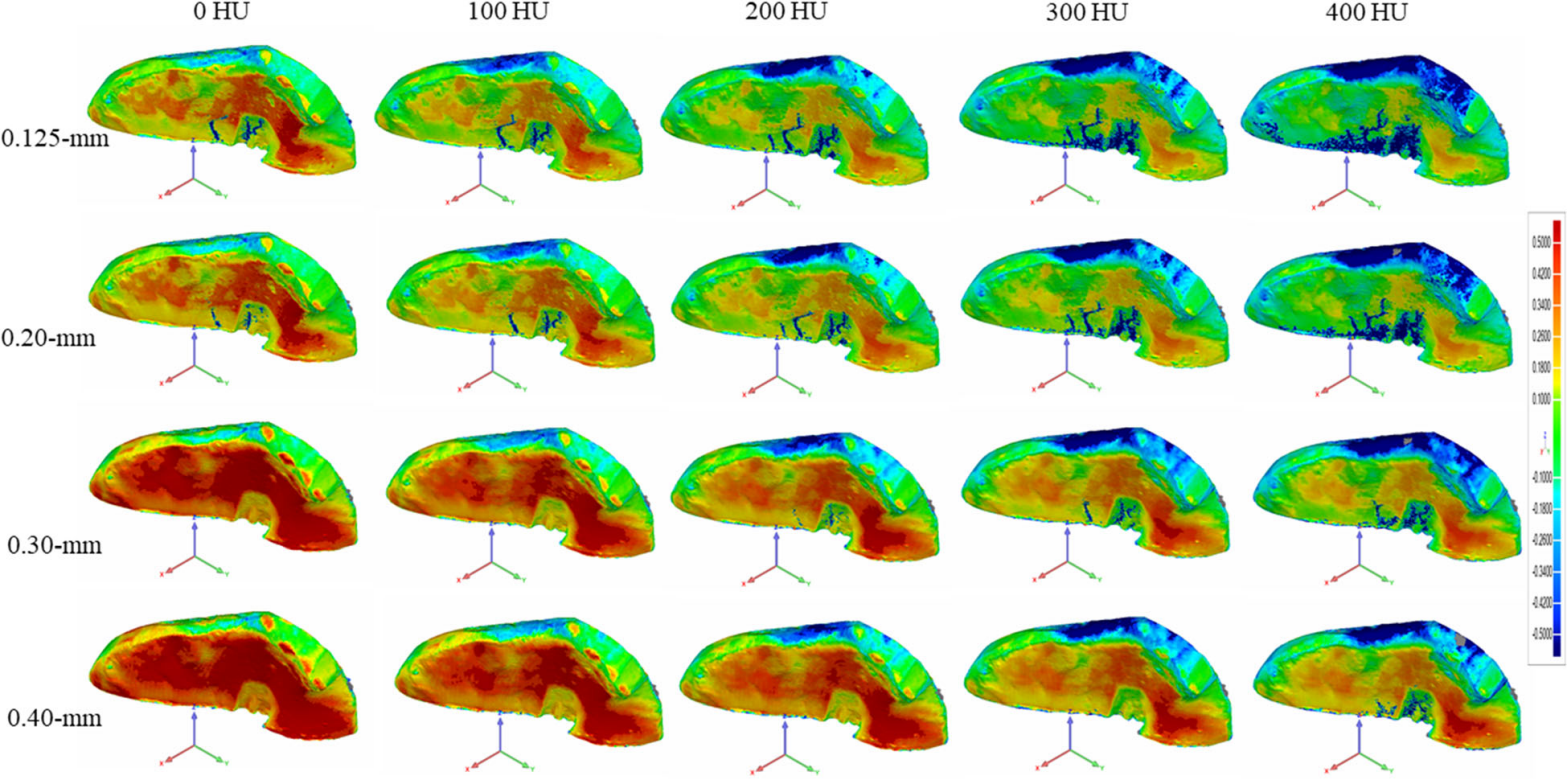
CBCT scanning register with laser scanning. Each CBCT scanning file of different voxel size (.125 mm, .20 mm, .30 mm, .40 mm) and different HU (0, 100, 200, 300, 400) was then individually superimposed on the laser scanning file (labeled as the reference file) by means of an automated best-fit algorithm. A .5-mm threshold parameter was set as the critical value to analyze deviations between the laser scanning file (reference file) and each CBCT scanning file (test file). The darker the color is, the larger the variance is, and the lighter the color is, the smaller the variance is. For the same HU value (column), with the increase of voxel size, the color becomes darker, showing the increase of the variance between the CBCT file and the laser file. For the voxel size (line), each voxel size has an optimal HU value with the lightest color (100 for .125 mm, 200 for .2 mm, 300 for .3 mm, and 400 for .4 mm)

**Fig. 2:**
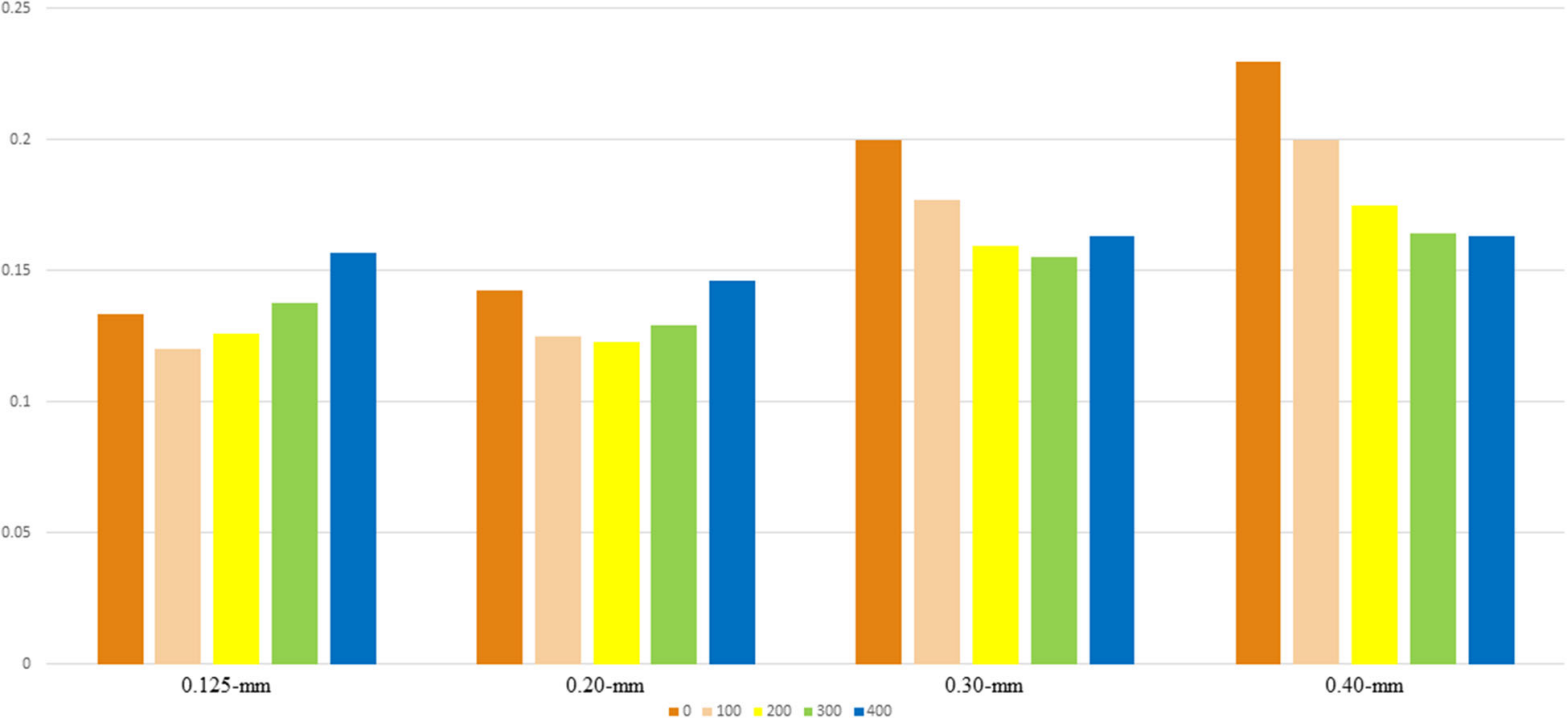
Mean iterative distance of the superimposition. The mean iterative distance of the superimposition of each CBCT scanning file and laser scanning file was auto-calculated. For the .125-mm voxel size, the least iterative distance was achieved when we chose 100 as the minimum HU threshold of segmentation. This equally applied to 200 for the .2-mm voxel size, 300 for the .3-mm voxel size, and 400 for the .4-mm voxel size as the minimum HU threshold of segmentation. Statistical significance can be seen in the .4-mm group (*P* = .07)

**Fig. 3:**
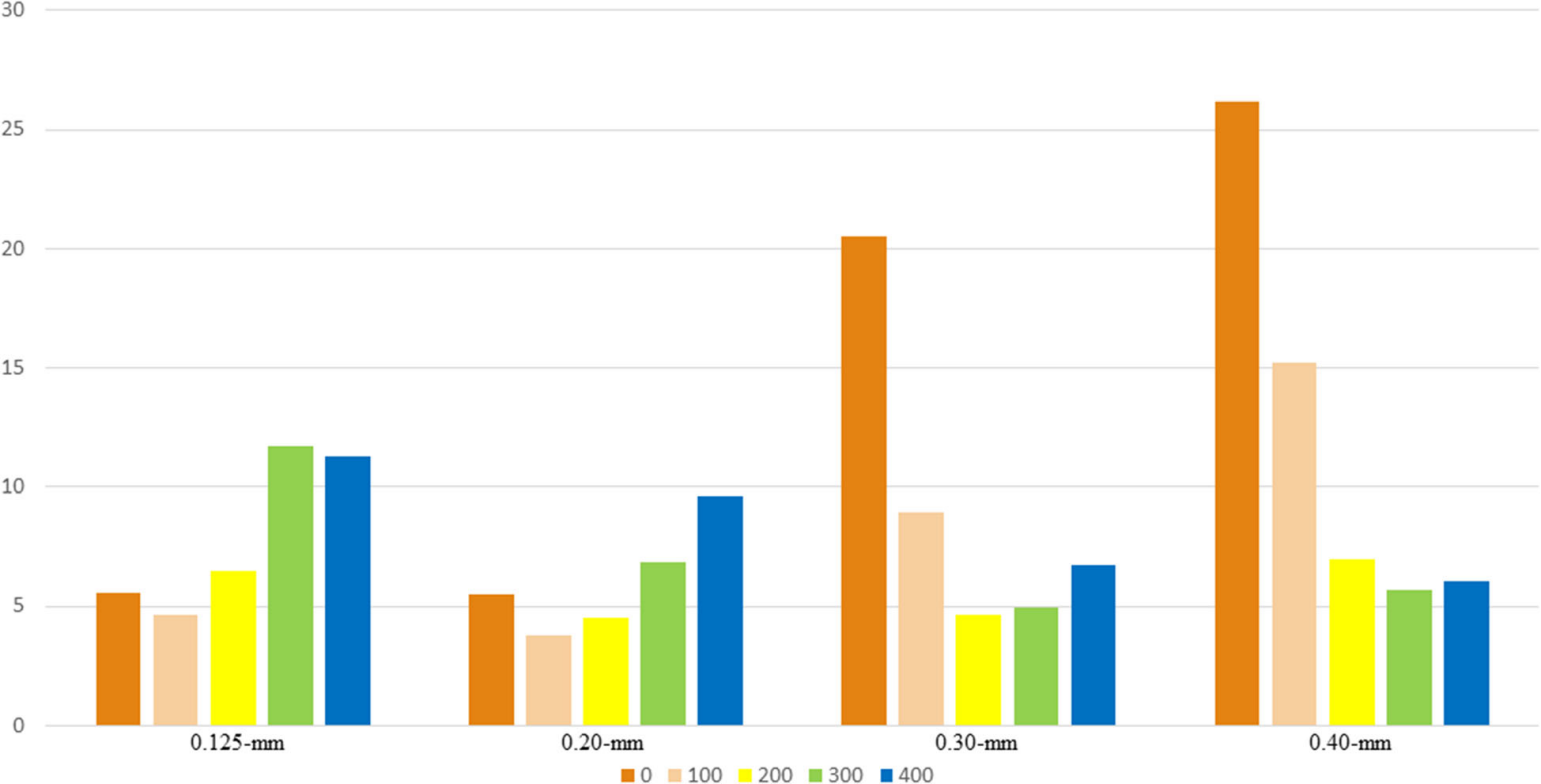
Mean total percentages of the points outside the bounds of the superimpositions. A 5-mm threshold parameter was set as the critical value to analyze deviations between the laser scanning file (reference file) and each CBCT scanning file (test file). Reports were generated for calculating the total positive and negative deviations separately. Seen as a whole, for the .125-mm voxel size, the lowest percentage was achieved when we chose 100 as the minimum HU threshold of segmentation. Similarly, 100 and 200 HU created the lowest percentage for the .20-mm voxel size, 200 and 300 HU created the lowest percentage for the .30-mm voxel size, and 300 and 400 HU created the lowest percentage for the .40-mm voxel size

All mandibular pieces were covered with double layers of boxing wax for soft-tissue simulation. CBCT acquisition was performed with a KaVo 3D exam scanner (KaVo Dental) with a field-of-view size (FOV) of 8.5 × 8.5 cm, 120 kv, and 5 mA. A foam pad with a flute was used to stabilize pieces of the mandible in our study. Each sample was scanned 4 times with 4 voxel sizes: .125 mm, .20 mm, .30 mm, and .40 mm. The CBCT scan parameters are presented in Table 1. After scanning was completed, the CBCT data were exported as DICOM format files and imported into Mimics software (version 10.01; Materialise, Leuven, Belgium) for segmentation and 3D reconstruction. The mandibular cavities were filled by the “cavity fill” tool in the Mimics software to obtain the intact volume. The minimum segmentation thresholds in HU were set as 0, 100, 200, 300, and 400, respectively, for each voxel size, and the maximum segmentation thresholds remained unchanged. The 3D models of CBCT scans were then imported as STL format files into Geomagic Control software (3D Systems) for volumetric measurement and registration. Each CBCT scanning file (labeled as the test file) was then individually superimposed on the laser scanning file (labeled as the reference file) by means of an automated best-fit algorithm. A color map was made for comparison of the mean iterative distance of the CBCT scan and the laser scan (Fig. 1). A .5-mm threshold parameter was set as the critical value for the analysis of deviations between the laser scanning file (reference file) and each CBCT scanning file (test file) (Fig. 2). Any points in the test file deviating from the reference file by more than .5 mm in the positive or negative direction were considered to be beyond the upper or lower limits, accordingly. Reports were generated for separate calculation of the total positive and negative deviations (Fig. 3).

**Table 1:** Preset CBCT scanning parameters

*Group*	*Voxel size (mm)*	*FOV (mm* ^*2*^ *)*	*Scan time (sec)*	*Tube current (mA)*	*Tube voltage (kV)*
CS 0.125	0.125	85*85	23	5	120
CS 0.2	0.20	85*85	23	5	120
CS 0.3	0.30	85*85	8.9	5	120
CS 0.4	0.40	85*85	8.9	5	120

*CS*, CBCT scan; *FOV*, field of view

IBM SPSS Statistics 20.0 (IBM, Chicago, IL, USA) was used for statistical analysis. Due to the different sizes of the mandibular pieces, the within-group variation was the major difference; thus, we chose a paired *t* test (each test file and reference file) to analyze the effect of the Hounsfield unit threshold of segmentation with different voxel sizes.

## Results

The means and standard deviations of the HU thresholds of water, air, and wax were 55.0 ± 10.8, − 980.0 ± 13.7, and − 270.0 ± 36.8, respectively. The means of the volume measurements of laser scans and CBCT scans are presented in Table 2. The measurements of CBCT-scan volumes deviated from those of laser-scan volumes by + 7.67% to − 3.05%, with different HU thresholds of segmentation and voxel sizes (Table 2). The deviation increased with the voxel size. For each voxel size, there was a more suitable HU threshold of segmentation that showed no significant difference from the laser scan (minimum 100 for .125 mm, 200 for .20 mm, 300 for .30 mm, and 400 for .40 mm). The other testing HU threshold of segmentation showed a significant difference from the reference (*P* < .05).

**Table 2:** Volumetric measurement of different CBCT voxel sizes with 5 minimum thresholds compared with laser scan

*Voxel size (mm)*	*HU*	*Mean vol (mm* ^*3*^ *)*	*Deviation%*	*P*
.125	0	24,436.83	1.91	.004*
	100	24,007.69	.12	.794
	200	23,690.75	−1.2	.018*
	300	23,350.4	−2.62	.000*
	400	23,014.19	−4.02	.000*
.20	0	24,695.64	2.99	.000*
	100	24,282.46	1.27	.020*
	200	23,959.73	−.07	.855
	300	23,601.82	−1.57	.009*
	400	23,246.79	−3.05	.000*
.30	0	25,494.24	6.32	.000*
	100	24,968.95	4.13	.000*
	200	24,506.46	2.2	.002*
	300	24,018.74	.17	.757
	400	23,604.68	−1.56	.018*
.40	0	25,817.22	7.67	.000*
	100	25,275.56	5.41	.000*
	200	24,771.32	3.31	.000*
	300	24,291.91	1.31	.044*
	400	23,898.76	−.33	.52
Laser		23,978.13		

*P < 0.05

The mean iterative distance of the superimposition of each CBCT scanning file and laser scanning file was auto-calculated by the Geomagic software, and a histogram was made to show the deviation visually (Fig. 2). For the .125-mm voxel size, the least iterative distance was achieved when we chose 100 as the minimum HU threshold of segmentation. This applied equally to 200 for the .20-mm voxel size, 300 for the .30-mm voxel size, and 400 for the .40-mm voxel size as the minimum HU threshold of segmentation. Statistical significance can be seen in the .40-mm group (*P* = .07). Seen as a whole, the mean iterative distance increased with the voxel size. Mean total percentages of the points outside the bounds of the superimpositions were also recorded (Fig. 3). For the .125-mm voxel size, the lowest percentage was achieved when we chose 100 as the minimum HU threshold of segmentation. Similarly, 100 and 200 HU created the lowest percentages for the .20-mm voxel size, 200 and 300 HU created the lowest percentage for the .30-mm voxel size, and 300 and 400 HU created the lowest percentage for the .40-mm voxel size.

## Discussion

The quality of images obtained by CBCT depends on many acquisition parameters such as tube voltage, tube current, the field of view (FOV), HU threshold of segmentation, and voxel size [9]. To control the above factors, we chose the same CBCT scanner, the same scanning tube voltage and tube current, and the same FOV. The main variables were voxel sizes (.125 mm to .40 mm) and HU threshold settings of segmentation and will be discussed below.

Voxel size is one of the critical parameters that influence the volumetric measurement of 3D-reconstructed jaws. Voxel size is the minimum unit of digital data segmentation in three-dimensional space, similar in concept to pixels in two-dimensional space. It is of paramount importance in terms of scanning and reconstruction times, as well as quality of CBCT images [11]. Ye et al. [10] discovered that the volume measurements of teeth tended to be larger with increasing voxel sizes during scanning (with laser scanning as the gold standard). Sang et al. [12] found that increasing voxel resolution from .30 to .15 mm did not result in increased accuracy of 3D tooth reconstruction (with 3Shape optical scanning as the gold standard). Hassan et al. [13] investigated the influence of voxel size on the quality of the 3D surface models of the dental arches from CBCT and found that large voxel size reduced the visibility of the occlusal surfaces and bone in the anterior region in both the maxilla and mandible.

Meanwhile, the HU threshold is also an important parameter in 3D reconstruction. Computed tomography uses HU — a numeric value that represents tissue density by quantitative measurement of tissue absorptivity for x-rays — as its unit of measure. Computed tomography values can be calculated as (μ_tissue_-μ_water_)*k/μ_water_, whereμ is the x-ray absorption coefficient of tissue, and k is the constant [10]. However, there is insufficient research on the influence of voxel size and reconstruction threshold on the accuracy of volumetric measurement of the mandible from CBCT. There is a significant difference among the HU values of CBCT obtained by different manufacturers. The CBCT in this study was calibrated based on each manufacturer’s instructions as the reference, and the HU values of CBCT we obtained were close to the CT values. Therefore, the results of this study can provide some reference to guide this kind of CBCT reconstruction.

The deviation of volumetric measurement may be due to the artefacts of CBCT scanning and 3D reconstruction. Artefacts are discrepancies between the reconstructed visual image and the actual physical image which degrade the quality of CBCT images [14], including extinction artefacts, beam-hardening artefacts, partial volume effect, ‘aliasing’ artefacts, ring artefacts, and motion artefacts, as well as noise and scatter [15]. In this study, we found that the mandibular volume measurements from the CBCT scans were larger than those from the laser scans with the increase of voxel sizes. This result might be owing to the surface-surrounding artefacts that can be induced by the partial-volume effect and scatter, which act as halation around the mandible. The partial-volume effect [10, 16] is a common artefact in computed tomography and can produce deviation in the digital image. According to the theory of the partial-volume effect, the value of each pixel (voxel size) on the CT image represents the average CT value of the corresponding unit. It cannot reflect the CT value of the diverse structures in the unit faithfully. Therefore, when we used a larger voxel size in our study, the volume of reconstructed bone was larger than its reality by artefacts. There is little research on the relationship of the HU threshold of segmentation and CBCT volumetric measurement.

In our study, we showed that with increased voxel size, the artefacts of CBCT scanning increased from .125 mm to .40 mm, and the volumetric measurement increased for each corresponding HU threshold of segmentation. For this reason, a smaller range of thresholds is required to diminish the increased artefacts. This explains why the optimal HU threshold of segmentation differs, to some extent, with different voxel sizes. The minimum threshold increases, the range of threshold narrows, and some artefacts may be hidden.

In this study, we chose laser scanning as the reference to compare the accuracy of CBCT volumetric measurement with different HU thresholds of segmentation and voxel sizes. Micro-computed tomography and laser scanning were often used as references in previous research. According to Teeter et al. [17], both the micro-computed tomography and laser scans produced complete reconstructions of the surfaces, and micro-computed tomography has superior repeatability compared with laser scanning (mean of 1 mm for micro-computed tomography vs 19 mm for laser scans). Micro-computed tomography requires a rather long time to scan and reconstruct, depending on the size of the object and scan resolution. Laser scanning, in contrast, requires a much shorter time, and its data are much more amenable to transmission and processing. Today, many scholars use laser scanning as the reference. Ye et al. [10] used laser scanning as the reference to study the accuracy of volumetric measurements of teeth in vitro by CBCT. Sang et al. [12] used laser scanning as the reference to assess the linear, volumetric, and geometric accuracy of 3D reconstructions from CBCT and to investigate the influence of voxel size and the CBCT system on the reconstruction results. Lemos et al. [18] evaluated the reliability of measurements made on digital cast models scanned in the 3Shape R700 scanner and found laser scanning to be reliable in producing a digital version of the physical models.

ALARA [19], the acronym used in radiation safety (“As Low As Reasonably Achievable”), requires that ionizing radiation be maintained as low as reasonably achievable, which means choosing the minimum resolution that does not affect diagnostic accuracy. Ionizing radiation (IR) is a known carcinogen and produces DNA damage directly or indirectly. A recent study reported that the dental pulp stem cells (DPSCs) exposure to CBCT induced transient DNA damage and persistent inflammatory reaction in DPSCs [20]. Images acquired in smaller voxel sizes exactly have better qualify, but also increase the radiation dose to the patient. There may be no significant difference in the diagnostic outcome compared with slightly lower resolution images within a certain range, so we must choose optimal voxel size based on the reliability and accuracy of the diagnostic outcome and radiation dose. Many of these parameters can be varied according to the diagnostic task, but no protocols have yet been established for specific diagnostic tasks in dentistry. This study paid specific attention to the optimal HU thresholds for different voxel sizes, which may provide a new avenue to a suitable protocol.

### Limits

In this research we found that voxel sizes and HU thresholds indeed have significant effects on the volumetric measurement of CBCT 3D reconstruction, which deserves more attention in our clinics. Clinicians need to choose suitable voxel size to do the scanning and optimal HU thresholds to do the reconstruction in order to improve the accuracy of volumetric measurement. In consideration of radiation dose, we adopted an in vitro experiment for preliminary exploration and chose pig mandibles as samples, which may cause some unavoidable divergence due to the anatomic differences between humans and pigs. Only one CBCT machine was adapted in this study and this may be a limitation of this research. Also, an in vitro experiment cannot completely reflect true clinical conditions, so an in vivo experiment is expected in future research. Larger sample sizes are also anticipated. Also, only one CBCT machine was adapted in this study and this may influence the validity of this research.

## Conclusions

Voxel sizes and Hounsfield unit thresholds influence the accuracy of volumetric measurements in CBCT scanning. The artifacts increase with the voxel size, and there exist optimal Hounsfield unit thresholds correspond to different voxel sizes. We are thus reminded to select optimal parameters to perform CBCT scanning and 3D reconstruction in clinical work.

## Data Availability

The datasets used and/or analysed during the current study are available from the corresponding author on reasonable request.

## Abbreviations

3D: Three-dimensional
CBCT: Cone-beam computed tomography
FOV: Field of view
HU: Hounsfield units
